# Optimal Sharpening of Compensated Comb Decimation Filters: Analysis and Design

**DOI:** 10.1155/2014/950860

**Published:** 2014-01-22

**Authors:** David Ernesto Troncoso Romero, Massimiliano Laddomada, Gordana Jovanovic Dolecek

**Affiliations:** ^1^Electronics Department, INAOE Institute, 72840 Tonantzintla, PUE, Mexico; ^2^Electrical Engineering Department, Texas A&M University, Texarkana, TX 75505, USA

## Abstract

Comb filters are a class of low-complexity filters especially useful for multistage decimation processes. However, the magnitude response of comb filters presents a droop in the passband region and low stopband attenuation, which is undesirable in many applications. In this work, it is shown that, for stringent magnitude specifications, sharpening compensated comb filters requires a lower-degree sharpening polynomial compared to sharpening comb filters without compensation, resulting in a solution with lower computational complexity. Using a simple three-addition compensator and an optimization-based derivation of sharpening polynomials, we introduce an effective low-complexity filtering scheme. Design examples are presented in order to show the performance improvement in terms of passband distortion and selectivity compared to other methods based on the traditional Kaiser-Hamming sharpening and the Chebyshev sharpening techniques recently introduced in the literature.

## 1. Introduction

Efficient decimation filtering for oversampled discrete-time signals is key in the development of low-power hardware platforms for reconfigurable communication transceivers [1–26]. From a practical point of view, decimation is usually accomplished using a cascade of two (or more) stages. The filter in the first stage is a comb filter of order *K* decimating by a factor *M*, with *z*-transfer function and zero-phase frequency response, respectively, given as

(1) $$\begin{array}{c} H^{K}\left(z\right)={\left(\frac{1}{M}\frac{1-z^{-M}}{1-z^{-1}}\right)}^{K}, \end{array}$$

(2) $$\begin{array}{c} H^{K}\left(\omega \right)={\left(\frac{1}{M}\frac{\sin\left(\omega M/2\right)}{\sin\left(\omega /2\right)}\right)}^{K}. \end{array}$$

Comb filters are used in the first stage of the decimation chain because their system function is simple and it does not require any multiplier. However, their magnitude response exhibits a considerable passband droop in the passband *Ω*_*p*_,

(3) $$\begin{array}{c} \Omega_{p}=\left[0,\omega_{p}\right],\;\;\omega_{p}=\frac{\pi }{D}=\frac{\pi }{M\upsilon }, \end{array}$$

where *D* = *Mυ* is the total decimation factor and *υ* is the residual decimation factor of the remaining stages in the multistage architecture. Furthermore, comb filters have low attenuation in the folding bands *Ω*_*k*_ defined as

(4) $$\begin{array}{c} \Omega_{k}=\left[\frac{2\pi k}{M}-\omega_{p},\frac{2\pi k}{M}+\omega_{p}\right],\;k=1,2,\ldots ,\left\lfloor \frac{M}{2}\right\rfloor , \end{array}$$

where ⌊*x*⌋ stands for the integer part of *x*.

Owing to their reduced computational complexity, research on comb filters to date has been focused on (1) improving the magnitude characteristic, (2) preserving linearity of phase, and (3) having the least possible increase of computational complexity [2–24]. With this background, let us review the literature in these three categories.

From the representative sample of works improving the magnitude characteristics of comb filters, we observe that the rotated-comb-based schemes [2–7] have the disadvantage of being susceptible to imperfect pole-zero cancelation. An effective way to prevent this problem consists in designing nonrecursive filters [3, 4, 7] with filtering implemented in polyphase form for ensuring power savings. However, this may result in higher demand for chip area. Other approaches improve the passband with low-order compensators and stopband attenuation by either increasing the order of the comb filter [8–14] or exploiting additional filtering at high rate [15–17]. These approaches provide low-complexity solutions, but the passband improvement cannot be completely controlled. Therefore, these methods are convenient when the desired magnitude characteristics are not too stringent, and when the bandwidth of interest is narrow.

On the other hand, the techniques relying on sharpening of comb-based filters in [18–24] are effective because they can take advantage of the structure proposed in [18], which harnesses the recursive form of comb filters resulting in Cascaded Integrator Comb- (CIC-) like architectures that move part of the filtering at lower rate. Additionally, this structure has all the sharpening coefficients at lower rate and, when integer coefficients scaled by a power of two are used, an effective overall structure is obtained, which does not suffer from finite-precision effects as rotated-comb-based methods.

Moreover, two-stage comb-based decimation schemes have gained great popularity because the comb decimation filter in the first stage, designed in nonrecursive form, can be implemented at lower rate by polyphase decomposition, thus resulting in lower power consumption. The second-stage filtering operates at lower rate as well, but it can take advantage of CIC-like architectures for area reduction. By doing so, the overall comb-based decimation scheme achieves power and area savings. This approach has been applied to traditional comb filters [25, 26] and to magnitude-improved comb filters [5, 15, 17, 20, 21, 24].

## 2. Problem Motivation, Contributions, and Paper Organization

The reasons at the very basis of this work stem from the following observations.

- Sharpened compensated comb filters [23, 24] based on the simplest polynomial of the traditional Kaiser-Hamming sharpening from [27] provide a good passband improvement over conventional comb filters. However, in methods [23, 24] the filter designer does not have control on the exact passband deviation and stopband attenuation achieved by the designed filter.
- In two-stage comb-based decimation schemes, magnitude response improvements over the passband and the first folding band can be achieved by improving only the second-stage comb filter. However, in these cases, the filter in the first stage introduces a passband droop that cannot be corrected neither by resorting to traditional Kaiser-Hamming sharpening [27] nor by using the recent Chebyshev sharpening [22] applied to the comb filter placed in the second stage. Thus, a different sharpening approach has to be pursued.

In the light of the previous observations, the contributions of this work are the following.

- We show that, for similar magnitude characteristics, sharpened compensated comb filters guarantee lower complexity than sharpened comb filters without compensation, especially when stringent specifications must be met.
- We introduce a low-complexity structure in which the simple multiplierless compensator can be embedded into the cascaded chain of comb filters working at lower rate.
- We detail the optimization framework to design sharpened comb-based filters to attain given specifications on the acceptable maximum passband distortion and selectivity. The optimized sharpening coefficients are finite-precision values resulting in multiplierless structures, which are important for low-power applications. The optimization problem can be straightforwardly solved with a simple routine of the MATLAB Optimization Toolbox (available online at [28]).

The rest of this paper is organized as follows.  Section 3 presents a summary of the generalized perspective of sharpening comb-based filters for decimation. The proposed filtering structure and the corresponding guidelines to decide when to use sharpened compensated filters instead of sharpened comb filters without compensation are introduced in Section 4. The optimization framework to design sharpened compensated comb filters along with the key design steps is provided in Section 5.  Section 6 highlights the characteristics to be considered for the sharpening of the second-stage filter in a two-stage comb-based architecture. Design examples are presented in Section 7 where the goal is to contrast the magnitude responses of the proposed method against existing techniques, namely, the ones based on traditional Kaiser and Hamming sharpening and the Chebyshev sharpening methods recently proposed in the literature. Comparisons in terms of computational complexity quantified in Additions Per Output Sample (APOS) are also included in that section. Finally, concluding remarks are presented in Section 8.

## 3. Generalized Perspective of Sharpening Comb-Based Filters for Decimation 

Let *F*(*z*) and *F*(*ω*) be, respectively, the transfer function and the zero-phase frequency response of an arbitrary comb-based filter to be sharpened (referred to hereafter as *subfilter*). Any arbitrary *N*th degree sharpening polynomial,

(5) $$\begin{array}{c} P\left(x\right)=\overset{N}{\underset{k=0}{\sum }}p_{k}x^{k}, \end{array}$$

allows mapping the amplitude values *x* = *F*(*ω*) to new amplitude values *y* = *P*(*x*). The new values *y* = *P*(*x*) must approximate the desired values *d* = *D*(*x*) for *x* ∈ *X*_*p*_ ∪ *X*_*s*_, where *X*_*p*_ = [*x*_*p*,*l*_, *x*_*p*,*u*_] and *X*_*s*_ = [*x*_*s*,*l*_, *x*_*s*,*u*_], with tolerances *δ*_*p*_ (for the approximation over the region *X*_*p*_) and *δ*_*s*_ (for the approximation over the region *X*_*s*_). In this way, the zero-phase frequency response of the sharpened filter achieves the desired values with a maximum absolute passband deviation *δ*_*p*_ over the range of *ω* where *F*(*ω*) ∈ *X*_*p*_ and a maximum absolute stopband deviation *δ*_*s*_ over the range of *ω* where *F*(*ω*) ∈ *X*_*s*_. Usually, *D*(*x*) = 1 for *x* ∈ *X*_*p*_ and *D*(*x*) = 0 for *x* ∈ *X*_*s*_. Thus, the sharpening polynomial must meet the following simultaneous conditions:

(6) $$\begin{array}{c} D\left(x\right)-\delta_{p}\leq P\left(x\right)\leq D\left(x\right)+\delta_{p},\;\text{for}\;x\in X_{p}=\left[x_{p,l},x_{p,u}\right],\\ D\left(x\right)-\delta_{s}\leq P\left(x\right)\leq D\left(x\right)+\delta_{s},\;\text{for}\;x\in X_{s}=\left[x_{s,l},x_{s,u}\right]. \end{array}$$

For the comb-based decimation filter, the range limits for *X*_*p*_ and *X*_*s*_ are

(7) $$\begin{array}{c} x_{p,l}={\min\left\{F(\omega )\right\}|}_{\omega \in \Omega_{p}},\\ x_{p,u}={\max\left\{F(\omega )\right\}|}_{\omega \in \Omega_{p}},\\ x_{s,l}={\min\left\{F(\omega )\right\}|}_{\omega \in \Omega_{1}},\\ x_{s,u}={\max\left\{F(\omega )\right\}|}_{\Omega_{1}}, \end{array}$$

where *Ω*_*p*_ and *Ω*_1_ are, respectively, given in (3) and (4).

## 4. Proposed Sharpened Compensated Comb Filters

Let us consider as subfilter the simplest compensated comb filter, which has the following transfer function [11]:

(8) $$\begin{array}{c} F\left(z\right)=H^{K}\left(z\right)\cdot \left\{-2^{-(b+2)}\left[1-\left(2^{b+2}+2\right)z^{-M}+z^{-2M}\right]\right\}. \end{array}$$

The zero-phase frequency response is

(9) $$\begin{array}{c} F\left(\omega \right)=H^{K}\left(\omega \right)\cdot \left(1+2^{-(b+1)}-2^{-(b+1)}\cos\left(M\omega \right)\right). \end{array}$$

The work [23] has shown that sharpening the subfilter *F*(*z*) with the polynomial *P*(*x*) = 2*x* − *x* ^2^ (a polynomial obtained using method [27]) results in significant improvement of the passband characteristic. In that method, the magnitude in the stopband regions can be arbitrarily improved with the order of the comb filter, *K*, and the parameter *b* must be adjusted accordingly. The polynomial *P*(*x*) = 2*x* − *x* ^2^ has been chosen in [23] because this is the simplest sharpening polynomial from [27] that can improve the passband. Due to that simplicity, it is inferred in [23] that the resulting sharpened compensated comb filter will have a low computational complexity.

In this paper, we propose to use the general sharpening polynomial from (5), finding the coefficients through optimization. The transfer function of the sharpened comb-based filter and its zero-phase frequency response are, respectively, given as

(10) $$\begin{array}{c} H_{SH}\left(z\right)=\overset{N}{\underset{k=0}{\sum }}z^{-(N-k)[K(M-1)/2+M]}p_{k}F^{k}\left(z\right),\\ H_{SH}\left(\omega \right)=\overset{N}{\underset{k=0}{\sum }}p_{k}F^{k}\left(\omega \right). \end{array}$$

**Figure 1:**
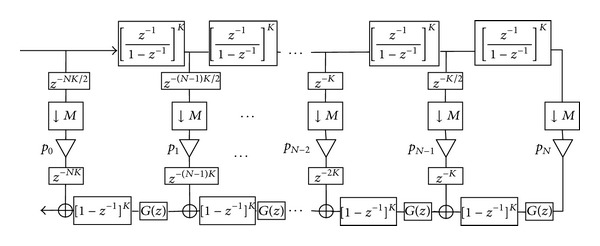
Proposed CIC-like structure for decimation filtering with sharpened compensated comb filters.

Figure 1 presents the proposed structure to efficiently implement a decimation filter in a CIC-like form. The structure is straightforwardly derived from the combination of both the structure from [18] and the structure introduced in [23] for the special case *P*(*x*) = 2*x* − *x* ^2^. Note that *K* must be an even value to avoid fractional delays.

The computational complexity of this structure measured in Additions Per Output Sample (APOS) is given by

(11) $$\begin{array}{c} A_{c}=N\left[K\left(M+1\right)+3\right]+Q-1+\overset{Q}{\underset{l=1}{\sum }}S\left(p_{k_{l}}\right), \end{array}$$

where *S*(*p*_*k*_*l*__) indicates the number of adders required to implement the sharpening coefficient *p*_*k*_*l*__ and *Q* is the number of nonzero sharpening coefficients. For comparison purposes, we present the computational complexity of a sharpening structure for comb filters without compensation (structure from [18]), which is given as

(12) $$\begin{array}{c} A=\tilde{N}K\left(M+1\right)+Q-1+\overset{Q}{\underset{l=1}{\sum }}S\left(p_{k_{l}}\right), \end{array}$$

with $\tilde{N}$ being the degree of the sharpening polynomial used in that structure.

From (11) and (12), we can see that the highest impact on the APOS complexity metric depends on the products *NK*[(*M* + 1) + 3] and $\tilde{N}K\left(M+1\right)$, respectively. Hence, *K* and *N* (or $\tilde{N}$) should be chosen as smaller values as possible for any arbitrary decimation factor *M*. Both *K* and *N* (or $\tilde{N}$) have the same impact on the APOS metric. However, *K* can only take even values, whereas *N* (or $\tilde{N}$) can also be odd. Since decreasing *K* is therefore more convenient, we can set *K* = 2 in advance. This choice leads us to use *b* = 1 in agreement with [23].

Let us discard the computational complexity introduced by the sharpening coefficients in both (11) and (12) and assume *K* = 2 for the reason discussed above. We will compare the terms *NK*[(*M* + 1) + 3] in (11) and $\tilde{N}K(M+1)$ in (12), assuming that $\tilde{N}=N+a$, with *a* being an integer. With this setup, we have

(13) $$\begin{array}{c} A_{c}\approx {\tilde{A}}_{c}={N\left[K\left(M+1\right)+3\right]|}_{K=2}=2N\left(M+1\right)+3N,\\ A\approx \tilde{A}={\tilde{N}K\left(M+1\right)|}_{K=2,\tilde{N}=N+a}=2N\left(M+1\right)+2a\left(M+1\right). \end{array}$$

Note that ${\tilde{A}}_{c}$ and $\tilde{A}$ differ in the terms 3*N* versus 2*a*(*M* + 1). Clearly, the proposed structure can have a lower computational complexity (i.e., ${\tilde{A}}_{c}<\tilde{A}$) when *a* > 0; that is, when $\tilde{N}>N$. In that case, sharpened compensated comb filters become convenient when

(14) $$\begin{array}{c} M>\left(\frac{3N}{2a}\right)-1. \end{array}$$

At this point, it is important to mention that we can take advantage of the frequency transformation approach [29] to estimate the minimum degree of the sharpening polynomial *P*(*x*) with any of the formulas from [30–32]. These formulas are expressible as *f*(*δ*_*p*_, *δ*_*s*_, *φ*_*p*_, *φ*_*s*_), where *δ*_*p*_ and *δ*_*s*_ are, respectively, the desired maximum absolute passband and stopband deviation of the sharpened filter, whereas *φ*_*p*_ and *φ*_*s*_ are given as [29]

(15) $$\begin{array}{c} \varphi_{p}=\cos^{-1}\left(\frac{2x_{p,l}-x_{p,u}-x_{s,l}}{x_{p,u}-x_{s,l}}\right),\\ \varphi_{s}=\cos^{-1}\left(\frac{2x_{s,u}-x_{p,u}-x_{s,l}}{x_{p,u}-x_{s,l}}\right), \end{array}$$

with *x*_*p*,*l*_, *x*_*p*,*u*_, *x*_*s*,*l*_, and *x*_*s*,*u*_ given in (7). Obviously, this is a preliminary estimation that depends on the accuracy of the formula being used. Substituting (7) in (15) and using the Kaiser formula [31], we can estimate *N* (or $\tilde{N}$) as

(16) $$\begin{array}{c} N\approx f\frac{\delta_{p},\delta_{s},\varphi_{p},\varphi_{s}}{2}=\frac{\left[-20\text{lo}{\text{g}}_{10}\left(\sqrt{\delta_{p}\delta_{s}}\right)-13\right]\pi }{14.6\left(\varphi_{s}-\varphi_{p}\right)}. \end{array}$$

**Figure 2:**
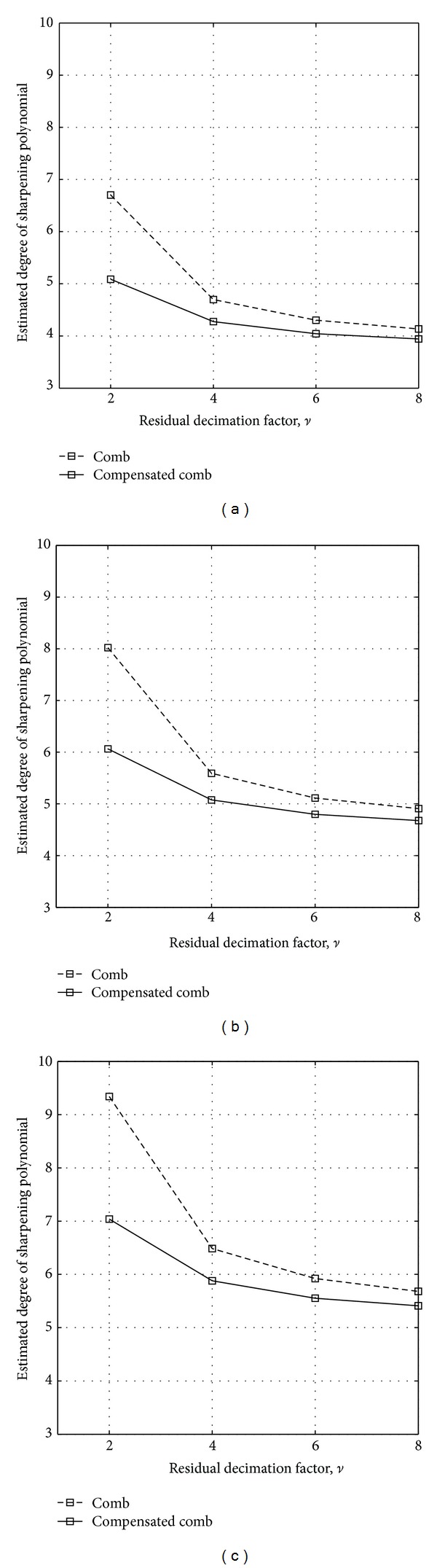
Estimated degree of the sharpening polynomial to sharpen comb filters (dashed line) and compensated comb filters (solid line), with *δ*_*p*_ = 0.001 and *δ*_*s*_ = 0.001 (a), *δ*_*s*_ = 0.0001 (b), and *δ*_*s*_ = 0.00001 (c).

Upon noticing that the shape of the magnitude response of comb filters changes very little with *M* [33], we set in advance *M* = 16 and we estimate the degrees *N* and $\tilde{N}$ using the Kaiser formula for some typical values of *υ*, *δ*_*p*_, and *δ*_*s*_; namely, *υ* = 2, 4, 6, and 8; *δ*_*p*_ = 0.001 (*≈*0.01 dB) and *δ*_*s*_ = 0.001 (60 dB), 0.0001 (80 dB) and 0.00001 (100 dB) (to estimate $\tilde{N}$, the values *x*_*p*,*l*_, *x*_*p*,*u*_, *x*_*s*,*l*_, and *x*_*s*,*u*_ must be obtained from (7) but first replacing *H* ^2^(*ω*) from (2) instead of *F*(*ω*) in these equations. To estimate *N*, we use *F*(*ω*) from (9) in these equations, first replacing *K* = 2 and *b* = 1 in (9)). These cases are shown in Figure 2. Note that, for these specifications, sharpened compensated comb filters are convenient when the residual decimation factor *υ* is equal to 2 or 4, that is, for small values. However, generally speaking, sharpened compensated comb filters become effective as the passband and stopband specifications become more stringent. From the previous analysis, we derive the following two important observations.

- In sharpened compensated comb filters, a lower computational complexity is obtained if *K* = 2. This is because both *K* and *N* have the same impact on the APOS metric. However, *K* can only take even values, whereas *N* can also be odd. Therefore, preserving a simple sharpening polynomial and improving the stopbands with the increase of *K*, as suggested in [23], do not guarantee a result with low computational complexity.
- Upon comparing the sharpened comb and sharpened compensated comb filters using *K* = 2, the former requires a polynomial with higher degree. As a consequence, its complexity is higher, despite the use of compensators in the latter. The reason is that the increased complexity in the sharpened compensated comb structures amounts to only 3 extra additions per polynomial degree (when the compensator from [11] is used), and these additions work at lower rate.

## 5. The Optimization Framework to Design Sharpened Comb Filters 

Now, we introduce the optimization framework to obtain the discrete coefficients of *P*(*x*) for which the maximum deviation of *P*(*x*) with respect to *D*(*x*), denoted by *δ*, is minimized. Note that this polynomial will attain the desired passband and stopband deviations with a proper polynomial degree. To find the sharpening polynomial coefficients, we evaluate the conditions (6) over a dense grid of points *x* covering the ranges *X*_*p*_ and *X*_*s*_.

Let us consider the following notation in order to formalize the optimization problem.

(i) *δ*_*p*_ and *δ*_*s*_ are the desired passband and stopband deviations after sharpening.
(ii) *m* = 10*N* is the number of points partitioning the frequency sets *X*_*p*_ and *X*_*s*_; that is, the overall number of grid points in the region *X*_*p*_ ∪ *X*_*s*_ is 20*N*.
(iii) ${\tilde{x}}_{i,p}$ and ${\tilde{x}}_{i,s}$ are the *i*th points belonging to the sets *X*_*p*_ and *X*_*s*_, respectively. To find these points, we divide the range of frequencies *Ω*_*p*_ = [0, *ω*_*p*_] into *m* equally spaced points ${\tilde{\omega }}_{i,p}$ and the range of frequencies *Ω*_1_ = [(2*π*/*M*) − *ω*_*p*_, (2*π*/*M*) + *ω*_*p*_] into *m* equally spaced points ${\tilde{\omega }}_{i,s}$, with *i* = 1, 2,…, *m*. Then, we set ${\tilde{x}}_{i,p}=F({\tilde{\omega }}_{i,p})$ and ${\tilde{x}}_{i,s}=F({\tilde{\omega }}_{i,s})$.
(iv) *d*_*i*,*p*_ and *d*_*i*,*s*_ are the desired amplitudes of the polynomial *P*(*x*) at the points $x={\tilde{x}}_{i,p}$ and $x={\tilde{x}}_{i,s}$, respectively. Usually, *d*_*i*,*p*_ = 1 and *d*_*i*,*s*_ = 0 for all *i*.
(v) [**M**]_*i*,*j*_ denotes the entry in the *i*th row and *j*th column of the underlined matrix **M**.
(vi) [**v**]_*i*_ denotes the *i*th element of the underlined vector **v**.
(vii) *B* is an arbitrary word-length for the fractional part in a fixed-point representation of the sharpening coefficients. In other words, every sharpening coefficient is an integer scaled by 2^−*B*^:
  (17) $$\begin{array}{c} p_{k}=2^{-B}{\tilde{p}}_{k},\;{\tilde{p}}_{k}\in \left\{\text{integers}\right\},\;0\leq k\leq N. \end{array}$$

By this setup, the optimization problem proposed in this paper can be written as

(18) min⁡s ⁡fTs  subject  to  As≤b,[s]i∈{integers},  2≤i≤N+2,

where **f** and **s** are vectors of size (*N* + 2) × 1, **A** is a matrix of size 4*m* × (*N* + 2) and **b** is a vector of size 4*m* × 1. In addition, we have

(19) $$\begin{array}{c} {\left[s\right]}_{i}=\{\begin{array}{cc} \delta ; & i=1,\\ {\tilde{p}}_{i-2}; & i=2,3,\ldots ,N+2, \end{array} \end{array}$$

(20) $$\begin{array}{c} {\left[f\right]}_{i}=\{\begin{array}{cc} 1; & i=1,\\ 0; & i=2,3,\ldots ,N+2, \end{array} \end{array}$$

(21) $$\begin{array}{c} {\left[A\right]}_{i,j}=\{\begin{array}{cc} -1; & 1\leq i\leq 2m,j=1,\\ -\frac{\delta_{s}}{\delta_{p}}; & 2m<i\leq 4m,j=1, \end{array} \end{array}$$

(22) $$\begin{array}{c} {\left[A\right]}_{i,j}=\{\begin{array}{cc} 2^{-B}{\left({\tilde{x}}_{i,p}\right)}^{j-2}; & 1\leq i\leq m,\;\;2\leq j\leq N+2,\\ -2^{-B}{\left({\tilde{x}}_{i-m,p}\right)}^{j-2}; & m<i\leq 2m,\;\;2\leq j\leq N+2,\\ 2^{-B}{\left({\tilde{x}}_{i-2m,s}\right)}^{j-2}; & 2m<i\leq 3m,\;\;2\leq j\leq N+2,\\ -2^{-B}{\left({\tilde{x}}_{i-3m,s}\right)}^{j-2}; & 3m<i\leq 4m,\;\;2\leq j\leq N+2, \end{array} \end{array}$$

(23) $$\begin{array}{c} {\left[b\right]}_{i}=\{\begin{array}{cc} d_{i,p}; & 1\leq i\leq m,\\ -d_{i-m,p}; & m<i\leq 2m,\\ d_{i-2m,s}; & 2m<i\leq 3m,\\ -d_{i-3m,s}; & 3m<i\leq 4m. \end{array} \end{array}$$

The optimization problem in (18) is a constrained mixed integer linear programming (MILP) problem whose solution can be obtained with generic MILP solvers. As Coleman pointed out in [22], these optimization resources could be inaccessible to many designers. However, the size of this problem is generally small and the simple MATLAB code available online [28] can be used straightforwardly. Such routine is based on the linprog function belonging to the MATLAB Optimization Toolbox. Once the vector **s** has been obtained, the sharpening coefficients can be found as follows:

(24) $$\begin{array}{c} p_{k}=2^{-B}{\left[s\right]}_{k+2},\;0\leq k\leq N. \end{array}$$

We notice in passing that a somewhat similar optimization approach was derived by Candan and made available online at [34], along with an extensive MATLAB code that, in general terms, finds the infinite-precision coefficients using the linprog function. However, the work [34] does not provide any method to find optimal discrete coefficients and simple rounding has been applied to the infinite precision solution, making pointless the infinite-precision optimization. Moreover, method [34] is focused on sharpening traditional comb filters without compensation.

### 5.1. Design Steps of the Proposed Method

Given the desired passband and stopband deviations *δ*_*p*_ and *δ*_*s*_, the design steps of the proposed method can be summarized as follows

1. Find *x*_*p*,*l*_, *x*_*p*,*u*_, *x*_*s*,*l*_, and *x*_*s*,*u*_ using (7). Then, find the values *φ*_*p*_ and *φ*_*s*_ with (15) and estimate the degree *N* of the sharpening polynomial using (16).
2. Obtain *m* = 10*N* equally spaced points ${\tilde{\omega }}_{i,p}$ and ${\tilde{\omega }}_{i,s}$, for *i* = 1, 2,…, *m*, over the regions *Ω*_*p*_ = [0, *ω*_*p*_] and *Ω*_1_ = [(2*π*/*M*) − *ω*_*p*_, (2*π*/*M*) + *ω*_*p*_], respectively, assigning ${\tilde{\omega }}_{1,p}=0$, ${\tilde{\omega }}_{m,p}=\omega_{p}$, ${\tilde{\omega }}_{1,s}=\left(2\pi /M\right)-\omega_{p}$, and ${\tilde{\omega }}_{m,s}=(2\pi /M)+\omega_{p}$, with *ω*_*p*_ given in (3). Then, set ${\tilde{x}}_{i,p}=F({\tilde{\omega }}_{i,p})$ and ${\tilde{x}}_{i,s}=F({\tilde{\omega }}_{i,s})$, with *F*(*ω*) given in (9).
3. Choose the desired word-length *B* and the desired values *d*_*i*,*p*_ and *d*_*i*,*s*_, for *i* = 1,2,…, *m*.
4. Create **f**, **A** and **b** using (20)–(23). Then, solve the problem (18) for **s**. A straightforward way is using the MATLAB routine available online at [28].
5. Obtain the sharpening coefficients *p*_*k*_ using (24).

## 6. Sharpening the Second-Stage Filter in a Two-Stage Architecture 

Earlier in this paper we pointed out that the two-stage comb-based structure, which can be formed when the decimation factor *M* can be expressed as *M* = *M*_1_ *M*_2_, is effective to balance area and power consumptions. When this structure is chosen, the second-stage comb filter must be carefully designed since this is the filter where the worst-case magnitude characteristic of the overall cascade does occur. Moreover, the first-stage comb filter introduces a passband droop that should be corrected as well. It is interesting to note that, with the proposed sharpening approach, we can obtain an overall magnitude response attaining desired passband and stopband deviations by improving only the second-stage filter. However, we must have monotonic magnitude characteristic over the passband region of the filter to be sharpened.

The transfer function of the proposed two-stage filter is

(25) $$\begin{array}{c} H_{TS}\left(z\right)=H_{1}^{K_{1}}\left(z\right)\cdot \left[\overset{N}{\underset{k=0}{\sum }}z^{-M_{1}(N-k)}p_{k}F_{2}^{k}\left(z^{M_{1}}\right)\right], \end{array}$$

where *H*_1_(*z*) = *H*(*z*), with *H*(*z*) given in (1) but replacing *M* by *M*_1_, and *F*_2_(*z*) = *F*(*z*), with *F*(*z*) given in (8) but replacing *M* by *M*_2_, *K* = 2, and *b* = 1. The zero-phase frequency response is

(26) $$\begin{array}{c} H_{T}\left(\omega \right)=H_{1}^{K_{1}}\left(\omega \right)\cdot \overset{N}{\underset{k=0}{\sum }}p_{k}F_{2}^{k}\left(M_{1}\omega \right), \end{array}$$

where *H*_1_(*ω*) = *H*(*ω*), with *H*(*ω*) given in (2) but replacing *M* by *M*_1_, and *F*_2_(*ω*) = *F*(*ω*), with *F*(*ω*) given in (9) but replacing *M* by *M*_2_, *K* = 2, and *b* = 1.

Let us identify

(27) $$\begin{array}{c} P\left(F_{2}\left(M_{1}\omega \right)\right)=\overset{N}{\underset{k=0}{\sum }}p_{k}F_{2}^{k}\left(M_{1}\omega \right). \end{array}$$

To correct the passband droop of the first-stage comb filter, whose zero-phase frequency response is *H*_1_^*K*_1_^(*ω*), the zero-phase frequency response of the second-stage sharpened filter, *P*(*F*_2_(*M*_1_ *ω*)), must be designed to follow an amplitude given by 1/*H*_1_^*K*_1_^(*ω*) over the frequency interval *Ω*_*p*_ = [0, *ω*_*p*_]. Therefore, the desired values *d*_*i*,*p*_ with *i* = 1,2,…, *m* must be chosen as

(28) $$\begin{array}{c} d_{i,p}=\frac{1}{H_{1}^{K_{1}}\left({\tilde{\omega }}_{i,p}\right)}, \end{array}$$

where ${\tilde{\omega }}_{i,p}$ is the *i*th point of the equally spaced partition of *Ω*_*p*_ (see Section 5). Since *H*_1_(*ω*) is monotonically decreasing over *Ω*_*p*_, all the desired values *d*_*i*,*p*_ are different from each other. Thus, $P({\tilde{x}}_{i,p})$ can approximate every value *d*_*i*,*p*_, with ${\tilde{x}}_{i,p}=F_{2}(M_{1}{\tilde{\omega }}_{i,p}$), if ${\tilde{x}}_{i,p}\neq {\tilde{x}}_{j,p}$ for all *i* ≠ *j*, *i*, *j* = 1, 2, …, *m*. To meet this condition, *F*_2_(*M*_1_ *ω*) must be monotonic over *Ω*_*p*_. Finally, the entries [**A**]_*i*,1_ and [**A**]_*i*+*m*,1_ in (21) must be multiplied by *d*_*i*,*p*_. Similarly, the entries [**A**]_*i*+2*m*,1_ and [**A**]_*i*+3*m*,1_ must be multiplied by $1/H_{1}^{K_{1}}({\tilde{\omega }}_{i,s})$, where ${\tilde{\omega }}_{i,s}$ is the *i*th point of the equally spaced partition of *Ω*_1_ = [(2*π*/*M*) − *ω*_*p*_, (2*π*/*M*) + *ω*_*p*_] (see Section 5). This is done in order to achieve an equiripple passband deviation in the overall filter *H*_*TS*_(*z*).

## 7. Examples and Discussion of Results 

The following examples are discussed to show the improvement of magnitude characteristics of comb filters achieved with the proposed method in comparison to other sharpening-based schemes recently introduced in the literature.

**Example 1 (see the example in Section 4 of [22])**

Consider *M* = 16, *υ* = 4, and *ω*_*p*_ = 0.907 *π*/(*Mυ*). The goal is to attain at least −100 dB gain in the folding bands, with an additional passband improvement without any specific constraint.

Let us consider the following solutions.

- A 1st-order comb filter (*K* = 1), presharpened by the polynomial *P*_1_(*x*) = 2*x* ^2^ − *x* ^4^ and then sharpened with the 5th-order degree, first kind, Chebyshev polynomial *P*(*x*) = 5*x* − 20*x* ^3^ + 16*x* ^5^ (solution using the presharpening approach introduced in Section 6 (a) of [22]). This filter is identified by *H*_*a*_(*z*).
- A compensated comb filter with *K* = 6 and *b* = −1, sharpened with the polynomial *P*(*x*) = 2*x* − *x* ^2^ (solution using the method of [23]). Let us call this filter *H*_*b*_(*z*).
- *H*_*c*_(*z*), a compensated comb filter with *K* = 2 and compensation parameter *b* = 1, sharpened with the polynomial *P*(*x*) = 2^−5^(−3*x* ^2^ + 131*x* ^3^ − 96*x* ^4^) (solution using the scheme proposed in this work with *δ*_*p*_ = 0.0006 and *δ*_*s*_ = 0.000032).

**Figure 3:**
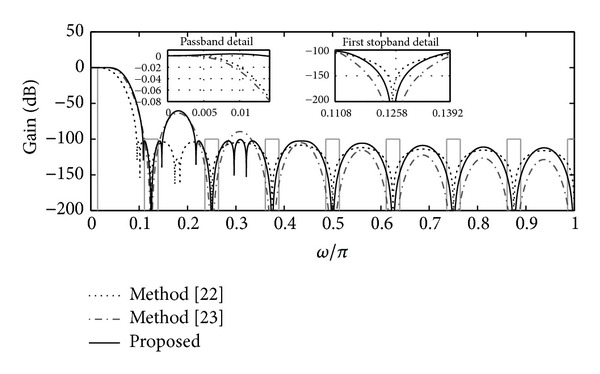
Magnitude responses of the filters *H*_*a*_(*z*) (method [22]), *H*_*b*_(*z*) (method [23]), and *H*_*c*_(*z*) (proposed method), presented in Example 1 with *M* = 16, *υ* = 4, and *ω*_*p*_ = 0.907 *π*/(*Mυ*).

Figure 3 shows the magnitude response of these filters, along with detail in passband and the first folding band. Notice that the three filters attain the −100 dB requirement in the folding bands. In the passband, the behaviors of filters proposed in [22, 23] are similar. The proposed filter, on the other hand, achieves better passband droop correction, which meets the 0.01 dB ripple (*δ*_*p*_ = 0.0006) specification.

When it comes to the complexities in terms of APOS, the proposed solution achieves better results too. The filter *H*_*a*_(*z*), implemented with a CIC-like structure, requires 20 integrators working at high rate, due to its double-sharpening scheme. Therefore, its APOS metric would be higher than 320 = 20 × 16. On the other hand, the APOS of *H*_*b*_(*z*) is 211 (see [23] for calculation of APOS in such structure). In the proposed method, we substitute *M* = 16, *K* = 2, *N* = 4, *Q* = 3, *S*(*p*_2_ = 3) = 1, *S*(*p*_3_ = 131) = 2, and *S*(*p*_4_ = 96) = 1 in (11) obtaining an APOS of 154. These results are summarized in Table 1.

**Example 2**

Consider a two-stage decimation filter with *M*_1_ = 8, *M*_2_ = 17, *υ* = 2, and *ω*_*p*_ = 0.9 *π*/(*M*_1_ *M*_2_ *υ*). The goal is to attain an attenuation of 60 dB in the folding bands with an additional passband improvement (without any given constraint). Assuming that the first-stage filter is a comb filter with *K*_1_ = 4, let us consider the following solutions for the second-stage filter:

- *H*_*a*_(*z*), a compensated comb filter with *K* = 6 and *b* = −1, sharpened with the polynomial *P*(*x*) = 2*x* − *x* ^2^ (solution using the method in [23]);
- *H*_*b*_(*z*), a compensated comb filter with *K* = 2 and compensation parameter *b* = 1, sharpened with the polynomial *P*(*x*) = 2^−5^(−*x* + 5*x* ^2^ + 116*x* ^3^ − 88*x* ^4^) (solution using the proposed scheme with *δ*_*p*_ = 0.0006 and *δ*_*s*_ = 0.001).

**Figure 4:**
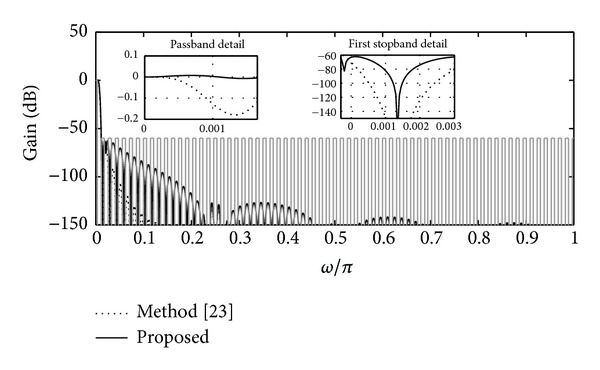
Magnitude responses of the filters *H*_*a*_(*z*) (method [23]) and *H*_*b*_(*z*) (proposed method), presented in Example 2 with *M* = *M*_1_ *M*_2_ = 8∗17 = 136, *υ* = 2, and *ω*_*p*_ = 0.9 *π*/(*Mυ*).

Figure 4 shows the magnitude response characteristics of these filters along with passband and first folding band details. Clearly, the filter designed with the proposed method presents both improvements: (1) better magnitude characteristic and (2) lower complexity, as summarized in Table 1. For this example, the APOS in Table 1 corresponds to the second-stage filter (the first-stage filtering is the same in both solutions and therefore it is omitted).

## 8. Conclusion

This paper proposed an optimization framework to design sharpening polynomials specifically suited to comb-based decimation filtering. The goal of the optimization problem was to minimize the min-max error over the frequency bands of interest of the sharpened filter. The optimization problem can be solved straightforwardly using the MATLAB Optimization Toolbox. The sharpening coefficients are guaranteed to be integers scaled by power-of-2 terms, thus resulting in low-complexity structures. Moreover, it was shown that the use of compensated comb filters, instead of combs only as basic building blocks in the sharpened filter, results in lower complexity structures (in terms of Additions Per Output Sample) for the same magnitude characteristics. Finally, it was shown that the proposed method provides better magnitude characteristic than other sharpening-based approaches for two-stage comb-based structures since it is able to correct the passband droop introduced by the first-stage comb filter.

## Figures and Tables

**Table 1:** Comparisons in terms of APOS for Examples 1 and 2.

Method	Example 1	Example 2
[22]	APOS > 360	—
[23]	APOS = 211	APOS = 223
Proposed	APOS = **154**	APOS = 164
